# The History of Therapeutic Aerosols: A Chronological Review

**DOI:** 10.1089/jamp.2016.1297

**Published:** 2017-02-01

**Authors:** Stephen W. Stein, Charles G. Thiel

**Affiliations:** ^1^ 3 M Drug Delivery Systems, St. Paul, Minnesota.; ^2^ Retired, Tucson, Arizona.

**Keywords:** atropine, dry powder inhaler, inhaler, metered dose inhaler, nebulizer, therapeutic aerosol

## Abstract

In 1956, Riker Laboratories, Inc., (now 3 M Drug Delivery Systems) introduced the first pressurized metered dose inhaler (MDI). In many respects, the introduction of the MDI marked the beginning of the modern pharmaceutical aerosol industry. The MDI was the first truly portable and convenient inhaler that effectively delivered drug to the lung and quickly gained widespread acceptance. Since 1956, the pharmaceutical aerosol industry has experienced dramatic growth. The signing of the Montreal Protocol in 1987 led to a surge in innovation that resulted in the diversification of inhaler technologies with significantly enhanced delivery efficiency, including modern MDIs, dry powder inhalers, and nebulizer systems. The innovative inhalers and drugs discovered by the pharmaceutical aerosol industry, particularly since 1956, have improved the quality of life of literally hundreds of millions of people. Yet, the delivery of therapeutic aerosols has a surprisingly rich history dating back more than 3500 years to ancient Egypt. The delivery of atropine and related compounds has been a crucial inhalation therapy throughout this period and the delivery of associated structural analogs remains an important therapy today. Over the centuries, discoveries from many cultures have advanced the delivery of therapeutic aerosols. For thousands of years, therapeutic aerosols were prepared by the patient or a physician with direct oversight of the patient using custom-made delivery systems. However, starting with the Industrial Revolution, advancements in manufacturing resulted in the bulk production of therapeutic aerosol delivery systems produced by people completely disconnected from contact with the patient. This trend continued and accelerated in the 20th century with the mass commercialization of modern pharmaceutical inhaler products. In this article, we will provide a summary of therapeutic aerosol delivery from ancient times to the present along with a look to the future. We hope that you will find this chronological summary intriguing and informative.

## The Delivery of Therapeutic Aerosols in Ancient Times

**FIG. 1.**
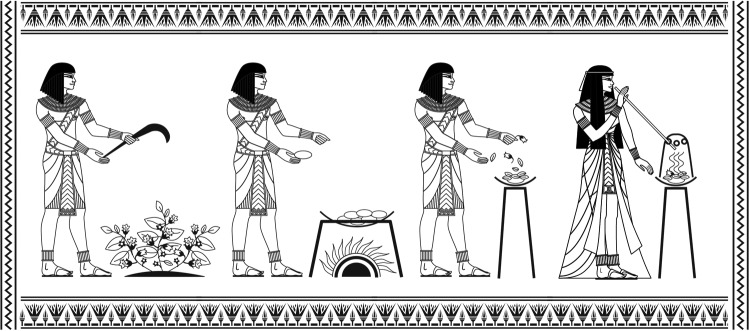
A schematic representation of the oldest known reference (c. 1554 BC) of therapeutic aerosol delivery in which smoke of henbane plants was inhaled through the stalk of a reed.

The delivery of therapeutic vapors and aerosols through inhalation has been used for thousands of years in various cultures. The first known reference to therapeutic aerosol delivery is an ancient Egyptian papyrus scroll (Ebers papyrus) dating back to ∼1554 BC, which purportedly was discovered between the legs of a mummy in the Assassif district of the Theban necropolis.^(1)^ This papyrus describes having patients struggling to breathe to inhale the vapor formed when black henbane (*Hyoscyamus niger*) plants were placed onto hot bricks. After placing the herbs onto the stone, a jar with a hole was place over the herbs and the patient inhaled the fumes through a stalk of reed that was placed into the hole. The instructions as translated by Ebbell^(2)^ are “Thou shalt fetch 7 stones and heat them by the fire, thou shalt take one thereof and place (a little) of these remedies on it and cover it with a new vessel whose bottom is perforated and place a stalk of a reed in this hole; thou shalt put thy mouth to this stalk so that thou inhalest the smoke of it.” Figure 1 shows a representation of the aerosol delivery described in the Ebers papyrus.

Black henbane is a leafy flowering plant native to Europe and northern Africa. The therapeutic properties of the inhaled vapor have been attributed to the tropane alkaloids, including atropine, contained in all parts of the henbane plant.^(3,4)^ The anticholinergic properties of atropine and structurally similar alkaloids combined with their prominence in various plants around the world have resulted in this class of compounds playing a critical role in the history of therapeutic aerosol delivery.

The most prominent ancient form of respiratory drug delivery was the smoking of opium for therapeutic and recreational purposes using incense burners and pipes. While the earliest known reference of smoking opium dates back to 1100 BC in China,^(5)^ the practice likely had earlier origins due to the prominence of opium cultivation by that time. It is believed that cultivation of the opium poppy originated among the Sumerian people of lower Mesopotamia and was passed on to the Assyrian and Babylonians who in turn passed the practice on to the Egyptians.^(5)^ By 1300 BC, opium cultivation had spread to Egypt, Carthage, and Europe. In 1025 AD, the Persian physician, Avicenna (Abū ‘Alī al-Husayn ibn Sina), in his influential medical encyclopedia entitled *Canon of Medicine*, described the use of opium for a variety of medical purposes, including analgesia, treatment of diarrhea, and treatment of severe cough. Avicenna described a variety of forms of administration, including smoking and nasal inhalation.^(6)^ Avicenna extensively described the toxicity of opioids and generally discouraged their use. He recommended, “If you have no other option but to use opioids, closely monitor patient's pulse to avoid overdosing.”^(6)^

The inhalation of therapeutic aerosols for the treatment of asthma is described in the writings of the influential Indian physicians, Charaka and Sushruta, which date back to ∼600 BC. These writings provide detailed instructions for preparing herbal compositions, including Datura that could be smoked in a pipe or in a cigarette^(3)^ to relieve asthma symptoms. Charaka also described the burning of herbal compositions in a bowl fitted with a second bowl on top and a tube fitted to allow inhalation of the fumes.^(7)^ The therapeutic effects and toxicity described by Sushruta are consistent with atropine, which is a known active ingredient of the Datura stramonium species.^(3)^ While the oldest existing documents describing smoking of Datura stramonium for treatment of asthma come from about 600 BC, Gandevia proposes that the origins of this therapy may date as far back as 2000 BC with early traditional Ayurvedic medicine.^(7)^

In addition to inhaled Datura, Indian physician Charaka Samhita describes in his first century AD medical book a range of asthma therapies that include steam inhalation and smoking cigars made of the paste of turmeric, cassia, cinnamon, the roots of the castor plant, lac, red arsenic, deodar, yellow orpiment, and nardus, smeared with ghee.^(8)^ The Ayurvedic texts provide instructions on how to modify the strength of the dose, give clear instructions on the optimal inhalation technique, and provide contraindications to this therapy.^(7)^

The famous Greek physician, Hippocrates (460–377 BC), describes a device for enabling the inhalation of various vapors for the treatment of a number of maladies. This device consisted of a pot with a lid that had a hole through which a reed could be placed to enable the vapors to be inhaled.^(9)^ Vapors generated from herbs and resins that had been boiled in vinegar and oil were inhaled through the reed.^(3)^

In the second century AD, the Greek physician, Galen of Pergamon, described the inhalation of powdered drugs for relief of nasal and chest troubles.^(10)^ In particular, Galen described the inhalation of myrrh and nutgall powders into the larynx through a bent reed to treat angina and credited the origin of this early practice of powder inhalation to Aesculapius, the God of medicine and healing.^(11)^ Around the same time frame, another Greek physician, Aretaeus of Cappadocia, utilized a similar instrument for powder inhalation to treat laryngeal ailments of children.^(12)^

While not normally delivered through inhalation, ephedra (known in China as Ma Huang) played a key role in the treatment of asthma for thousands of years. The Chinese medial book *Nei Ching* written by Huang-Ti in ∼1000 BC describes the use of Ma Huang remedies for the treatment of asthma.^(13)^ Ma Huang, which was usually delivered orally as a tea or a pill, was later shown to contain the active ingredient ephedrine.^(3)^ Ephedra was a mainstay in asthma therapy in the Roman Empire. The noted Roman historian, Pliny the Elder (23–79 AD), recommended ephedra mixed with red wine as a remedy for asthma. Interestingly, Pliny recommended a number of other asthma remedies that probably did not significantly advance the treatment of asthma (e.g., drinking the blood of wild horses, bear's gall mixed with water, or millipedes soaked in honey!), but he made the significant contribution of identifying a link between pollen exposure and respiratory distress [*The Natural History of Pliny*; translated in 1856 by Bostock and Riley^(14)^].

Ephedrine, which would later be isolated from ephedra by Japanese chemist Nagayoshi Nagai in 1885,^(15)^ remained widely used for treatment of cough and respiratory disease until the 1950s when it began to be displaced by other bronchodilators with improved safety profiles.^(15)^ Ephedrine sulfate is still available over the counter in the United States, but with significant restrictions and regulation.

By the first century AD, the smoking of tobacco and other plants in Central and South American cultures using ornate pipes had become common.^(3)^ It is believed that these cultures also had identified the smoking of Datura as a therapeutic remedy for the treatment of asthma^(7)^ and had used the inhalation of cannabis for recreational and therapeutic purposes, including the use as a sedative.^(10)^

In the fourth or fifth century AD, the Roman physician, Caelius Aurelianus, provided a clear description of the symptoms of bronchial asthma and proposed the inhalation of steam as a technique to treat asthmatic episodes. He also proposed inhaling sea air as a technique for preventing episodes.^(8)^ The warm steamy air in Roman public bath systems that were widely developed at that time in major cities was recommended by Roman physicians for treatment of various ailments, including asthma.^(10)^

## Delivery of Therapeutic Aerosols from the Middle Ages to the Industrial Revolution (476–1760 AD)

There were relatively few major innovations in the delivery of therapeutic aerosols during the period between the fall of Rome (476 AD) and the start of the Industrial Revolution (c. 1760 AD). The recorded examples of the delivery of therapeutic vapors and aerosols through inhalation during this period relied heavily on practices developed before the fifth century AD, such as the smoking of Datura or opium and directing the fumes and vapors of burning herbs into the lung of the patient.

The seventh century AD Greek physician, Paulus Aegineta, catalogued a host of ingredients to be inhaled for the treatment of persistent cough. His recommended treatment consisted of placing a complex herbal remedy onto coals and inhaling the fumes through a funnel. Aegineta's instructions are recorded as, “To be inhaled for a continued cough: storax, pepper, mastic, Macedonian parsley, of each one ounce; sandarach, 6 scruples; 2 bayberries; mix with honey; and fumigate by throwing them upon coals so that the person affected with the cough may inhale the vapor through a funnel.”^(9,16)^ The resultant vapor contained arsenic since sandarac is the Greek and Roman name for red arsenic sulfide.^(9,16)^

Rhazes, the Arab physician who lived in Baghdad from 850 to 932 AD, proposed some of the more innovative approaches for pulmonary delivery during the Middle Ages. He utilized sponges that had been soaked in a solution of narcotic plants (opium, hyoscyamus, mandrake, and henbane) and then allowed to dry. Then, just before the surgery, the sponge was moistened and placed over the mouth and nose of patient in order that the vapors be inhaled as anesthesia during surgery.^(10)^ Rhazes also advocated for the inhalation of arsenic for the treatment of respiratory conditions.^(3)^

The figure with the greatest influence on the inhalation of therapeutic aerosols during the Middle Ages was the Spanish-born physician, Maimonides (1138–1204 AD), who fled Spain and eventually became the personal physician to Saladin, the sultan of Egypt (1137 or 1138–1193 AD). Maimonides was responsible to care for the king's asthmatic son and wrote the first known book on asthma (*A Treatise on Asthma*) in 1190. His recommendations for management of asthma included inhaling herbs burned on a fire, abstaining from sex, and eating chicken soup.^(3)^ Maimonides provided numerous other dietary recommendations for the management of asthma and recognized the link between air pollution and asthma.^(13)^ Maimonides reasoned, “Town air is stagnant, turbid, and thick… Air winds carry stealthily inside the houses and many become ill with asthma without noticing. Concern for clean air is a foremost rule in preserving the health of one's body and soul” [quoted in Brenner^(13)^].

**FIG. 2.**
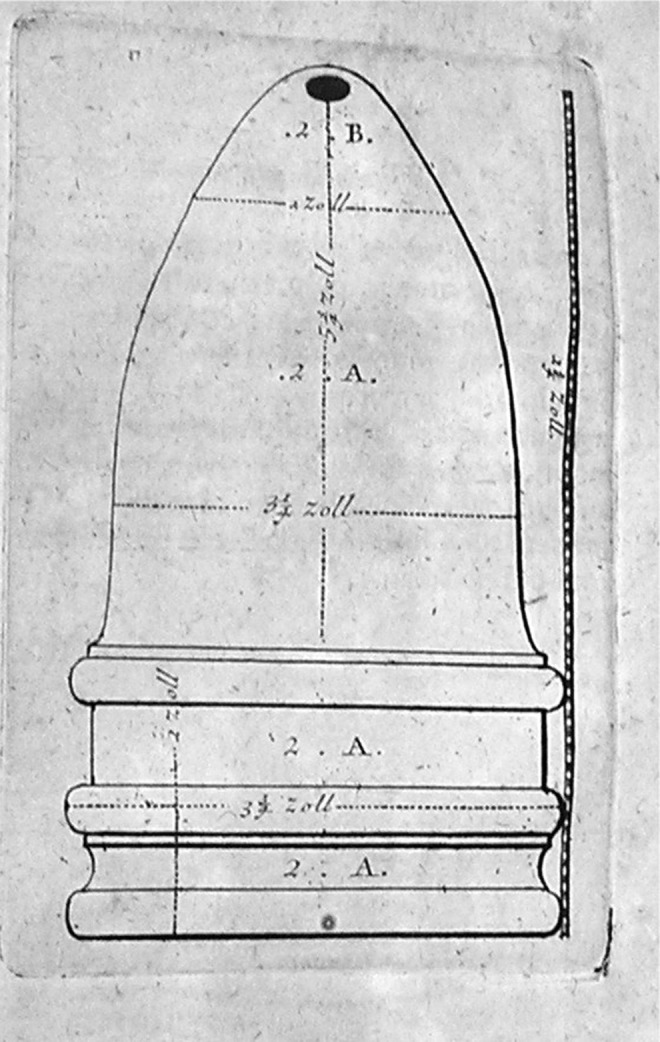
The oldest known drawing of a therapeutic inhaler device developed in 1654 by the English physician Christopher Bennet. Image courtesy of Mark Sanders.

There were limited advancements in the understanding of asthma and the delivery of therapeutic aerosols between the time of Maimonides and the start of the Industrial Revolution. The most notable contributions came from the Indian physician, Yogaratnakara, who in the 17th century provided further description and modification of Datura smoking therapy for treatment of asthma^(7)^ and English physician, Christopher Bennet, whose 1654 drawing (Fig. 2) is the oldest known illustration of an inhalation device.^(3)^

## The Delivery of Therapeutic Aerosols from 1760 to 1955

With the dawn of the industrial revolution in 1760, new manufacturing capabilities and technical discoveries paved the way for significant advances in the delivery of therapeutic aerosols. Before this time frame, therapeutic aerosols were primarily prepared directly by the physician or patient, but during this era, devices and therapeutic aerosol preparations began to be mass produced by individuals completely removed from the treatment of the patient. In addition, this was an era where new therapeutic entities were identified and techniques were developed for enhancing the potency and safety of these therapeutic entities by isolating the active ingredient. New delivery systems such as nebulizers and early dry powder inhalers (DPIs) were also introduced during this period. These advances set the stage for the beginning of the modern era of pharmaceutical aerosols, which began in 1956.

### Advances in the delivery of medicated vapors

In the late 1700s, respiratory drug delivery continued to rely on inhaling medicated vapors. It should be noted that the phrase “medicated vapor” is probably simplistic since some of the techniques described below that were utilized during this period undoubtedly resulted in some aerosol droplets being formed either through condensation of saturated water vapor in the system or through atomization of the medicated solution (e.g., when air is bubbled through the solution or the solution is boiled). As a result, the therapeutic benefit of these techniques was likely a result of both the vapor and aerosolized droplets. Nevertheless, these therapeutic aerosols were commonly referred to as medicated vapors and are similarly described in this article.

**FIG. 3.**
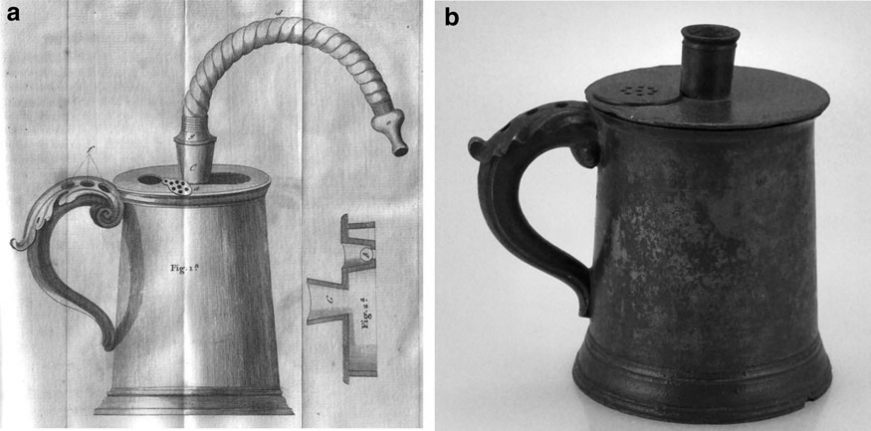
A drawing **(a)** and photo **(b)** of the Mudge Inhaler.^(17)^ As the patient inhaled through the flexible mouthpiece, air was drawn through the three holes on the handle and the air was bubbled through the medicated liquid before exiting the mouthpiece. The right side of the drawing shows the valve configuration, which utilized a small cork that moved and allowed the exhaled breath of the patient to exit the tankard. Images courtesy of Mark Sanders.

In his 1764 book, *Medical Advice to the Consumptive and Asthmatic Peoples of England*, English physician Philip Stern prescribed his personal recipe for inhalation of balsamic vapors for the treatment of asthma. Stern's book was groundbreaking in that it was intended to provide instruction for the general public rather than physicians.^(3)^ English physician John Mudge advocated for inhaling the aerosol from heated water containing opium for the treatment of catarrhal cough.^(3,9)^ In his book, *A Radical and Expeditious Cure for a Recent Catarrhous Cough*, he coined the term inhaler to describe a clever inhalation device for generating and delivering steam-based aerosols.^(17)^ The inhaler device, first introduced in 1778, consisted of a pewter tankard having a volume of approximately one pint with a lid that had a cover on the top with an adapter that could be coupled to a 5- or 6-inch-long flexible tube through which the patient inhaled for the 20–30-minute duration of the dosing (Fig. 3). The device had holes in the handle through which air was drawn in and bubbled through the warm liquid.^(17)^ Through use of a clever valve design, the patients were able to keep their lips surrounding the mouthpiece tube and breathe in and out through the tube in a way similar to many modern nebulizers. The Mudge inhaler marked the first known commercialization of an inhaler device with Mudge detailing in his book the name of a local pewterer that he partnered with and from whom the inhaler could be obtained.

Throughout the 1800s, the inhalation of medicated vapor from aqueous solutions continued to be a primary mode of treatment of respiratory ailments. Various ceramic inhalers with similar functionality to the Mudge inhaler were commercialized for generated medicated vapors and gained popularity in England in the 19th century. Prominent among these ceramic inhalers was the Nelson inhaler, which was commercialized by S Maw and Sons in London.^(9)^ Other approaches were used to generate medicated vapors, such as pouring the medicated solution over a sponge.^(18)^

As devices for generating medicated vapors proliferated, so too did the recommended therapies. In 1834, Sir Charles Scudamore proposed heating iodine and hemlock (conium) in water to 120 F and having the patient inhale the vapor for 15–20 minutes three times per day for the treatment of tuberculosis or other lung diseases.^(3)^ The 1867 British Pharmacopoeia listed detailed instructions for the generation of various medicated solutions to be inhaled, including hydrocyanic acid for the treatment of cough, chlorine for treatment of tuberculosis, hemlock for the treatment of cough, creasote for the treatment of tuberculosis and bronchitis, and iodine for the treatment of tuberculosis, pharyngitis, and laryngitis. The inclusion of these therapies in the British Pharmacopoeia demonstrates that these therapies had received widespread acceptance by that time.^(19)^

The inhalation of anesthetic gases through makeshift face masks emerged in the 1840s.^(10)^ There is a debate over whether the use of inhaled ether as surgical anesthetic was introduced by American physicians Crawford Long in 1842 or William Morton and John Collin Warren in 1846, but the practice gained rapid acceptance.^(3,20,21)^ Due to the side effect of nausea associated with ether, physician James Young Simpson introduced inhaled chloroform as a surgical anesthetic in 1847.^(10)^ The use of nitrous oxide for dental anesthesia was first demonstrated by American dentist Horace Wales in several dental operations in 1844^(10)^ and then gained widespread acceptance due to the influence of New York dentist Gardner Qunicy Colton.^(22)^

### The diversification of technologies for inhalation delivery in the last half of the 19th century

The last half of the 19th century saw unprecedented innovation in the area of pharmaceutical aerosol delivery technologies. The introduction of nebulizers, DPIs, advances in the commercialization of asthma cigarettes, and a number of other delivery technologies dramatically reshaped the practice of delivering drugs to the respiratory tract. Other innovations, although less influential, are worthy of mention. One innovation was direct spraying of medicated solutions into the respiratory tract. By 1852, Ira Warren (the inventor of the first DPI) was selling a kit consisting of a laryngeal, pharyngeal, and nasal shower syringe for applying an aqueous solution of silver nitrate for treatment of various respiratory conditions such as nasal catarrh and diseases of the throat.^(23)^ At the 1890 Annual Meeting of the American Medical Association, J. Mount Bleyer published an article describing a similar approach that he claimed was capable of administering medications such as silver nitrate, iodine, tannic acid, and hydrogen peroxide deeper into the bronchia.^(24)^

Inhaling the fumes of burning niter paper (which generates ammonia as it burns) was recommended by Henry Hyde Salter in his famous 1860 book, *On Asthma its Pathology and Treatment*, as a form of inhalation therapy.^(3)^ In the 1890s, the Wyeth Pencil Inhaler was commercialized as a portable and convenient treatment of various ailments, including catarrh, bronchitis, and croup. This inhaler contained menthol crystals and a rotatable cap with holes, which when in the proper orientation allowed air to penetrate through the holes and over the crystals to vaporize the menthol (which has a vapor pressure of 8.5 Pa at 25°C) so as to allow the vapor to be inhaled by the patient.^(25)^

Another interesting innovation during this period was a patent by Helbing and Pertch^(26)^ in 1899 of a propellant-based liquid aerosol generator that used ethyl or methyl chloride (now considered toxic via inhalation) as the propellant to atomize the liquid. The invention utilized heat of the hand to increase the vapor pressure of these liquids (135 and 506 kPa at 20°C, respectively) and provided sufficient pressure to atomize the formulation through a small orifice. Clark^(27)^ points out that the Helbing and Pertch inhaler was in many respects a precursor to the pressurized metered dose inhaler (MDI) that would reshape the treatment of lung diseases when introduced in 1956. However, Helbing and Pertch did not recognize the value of this invention for inhalation therapy and instead targeted applications requiring a medicated spray to be applied during surgery.

### Asthma cigarettes

**FIG. 4.**
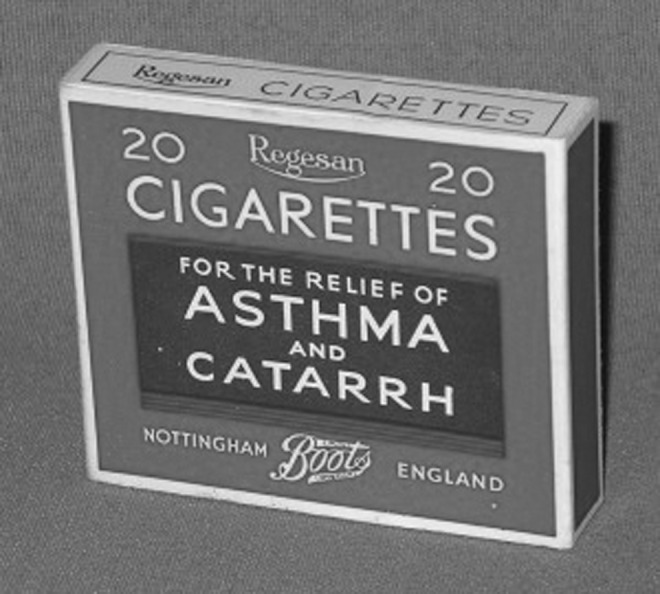
Page's asthma cigarettes containing stramonium, tea leaves, chestnut leaves, and gum benzoin. Image courtesy of Mark Sanders.

Smoke therapies for the treatment of respiratory ailments originated in India and date back to at least 600 BC. After being introduced in the United States by Philadelphia physician Samuel Cooper in 1797 and in Great Britain in 1802 by General Gent upon his return from India,^(28)^ smoked stramonium rapidly became a popular asthma therapy in the 1800s in Europe and the United States. Traditionally, these therapies were individually assembled for the specific patient (either by the patient or a physician). However, at the turn of the 20th century, there was a change to large-scale commercial manufacturing of cigarettes to be sold to unknown patients. A number of commercially available asthma cigarettes with stramonium were widely used in Europe, the United States, and China^(28,29)^ (Fig. 4). Some of the cigarettes included other herbs such as tea leaves, kola nuts, lobelia, and atropine-containing atropa belladonna leaves.^(9)^

Given the abundance of data demonstrating health risks associated with cigarette smoking, it may seem surprising to the 21^st^ century reader that cigarettes would be a preferred therapeutic option for patients with lung ailments. Clearly, the smoke from asthma cigarettes contained tar and a number of other toxic substances that would prove detrimental to lung function. However, the detrimental impact on lung function of cigarette smoking occurs gradually over time and is most severe with long-term use.^(30)^ On the other hand, the therapeutic impact of the atropine-containing smoke from cigarettes was significant and relatively rapid.^(31)^

The anticholinergic drug atropine is an extremely effective drug for treating asthma and chronic obstructive pulmonary disease (COPD). Atropine and its structural analog drugs, ipratropium and tiotropium, remain critical components of asthma and COPD therapy with annual sales in 2014 of greater than $7 billion.^(32)^ In addition, the extrafine nature of combustion aerosols such as cigarette smoke^(33)^ results in exceptional lung deposition that was likely significantly superior to the lung deposition provided by other delivery systems in the first half of the 20th century. As a result, the therapeutic benefit provided by asthma cigarettes may have significantly outweighed their potential harm.

### The emergence of atomizers and nebulizers

A significant advancement in the delivery of therapeutic aerosol was the invention and refinement of devices that reduce a medicated liquid to fine droplets for inhalation. These devices can be categorized into atomizers and nebulizers. Atomizer devices can use various approaches to cause the liquid to be broken into fine particles, but lack the baffle system of later nebulizer devices and generated coarse aerosol sprays of which only a small portion of the droplets that were small enough to deposit in the lung.^(19)^ Nebulizer devices are atomizers that contain a baffle system to remove coarse droplets from the air stream^(9,34)^ and thus provide aerosols that are more likely to deposit in the lung. Often the formulation contained in the large droplets that impact on the baffle falls back into the reservoir to be atomized again.^(19)^

**FIG. 5.**
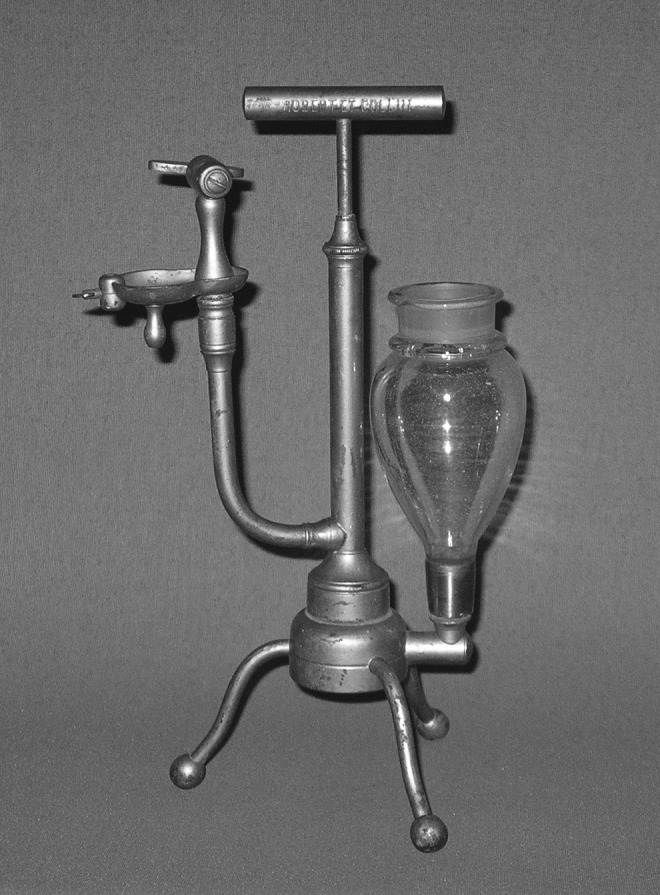
The pulverisateur developed by Jean Sales-Girons in 1858. Image courtesy of Mark Sanders.

The first atomizer device was developed in 1849 in France by Dr. Auphon in which he directed a jet of the water at a mineral spring against the walls of the Spa at Euzet Les Baines to break the liquid into fine droplets to be inhaled.^(9,35)^ In 1858, Jean Sales-Girons invented a portable atomizer that utilized a pump handle to draw liquid solution from a reservoir and atomize it through a small nozzle and direct it toward an impaction plate to produce a fine spray.^(19)^ Sale-Giron's nebulizer, called the pulverisateur, is shown in Figure 5.

In 1862, the German physician, Bergson, developed a different approach to break liquid into fine droplets. His device, called Hydrokonium, was an air jet atomizer in which a high-velocity air jet generated using a rubber squeeze bulb passed directly over another tube through which liquid was drawn up and atomized.^(19)^ This air jet atomizer approach, which would be widely applied on many other atomizers and nebulizers, applied the principle described in 1738 by the Swiss physicist Daniel Bernoulli and leveraged by Italian physicist Giovanni Battista Venturi that showed that suction could be obtained on a tube when a high-velocity fluid was placed directly over the tube.^(19)^

Atomizers and nebulizers rapidly became a key therapeutic option for treatment of a wide range of respiratory diseases. The rapid popularity is demonstrated by number of devices described in two key books from 1867 [Jacob Solis-Cohen^(11)^ and John M. Scudder^(36)^]. These two books provide excellent summaries of the state of the art in terms of atomizers and nebulizers at that time. They describe a number of novel devices that utilize a venturi principle to atomize medicated solutions using high-velocity airflow. These devices used a variety of sources for providing the pressurized airflow to atomize the liquid. In addition to Bergson's Hydrokonium, which utilized foot bellows to provide the airflow, Solis-Cohen described Siegle's steam atomizer, which utilized fire to create steam as the source of pressurized airflow.^(11)^ Atomizer designs based on Siegle's fire-powered steam nebulizer became popular in Europe, the United States, and Japan throughout the late 1800s.^(19)^ Early nebulizer systems with baffles are described in 1862 by the German physician, L. Waldenburg, and in Solis-Cohen's book published in 1867.^(11,19)^

At the dawn of the 20th century, improvements in the manufacturing of rubber allowed for the development of convenient, portable glass nebulizers that were powered by rubber squeeze bulbs.^(19)^ In the early 1930s, a nebulizer, the Pneumostat, using compressed air to power the atomization, was developed in Germany. Other compressed gas-powered nebulizers followed shortly thereafter.^(9)^ The first plastic nebulizer, the Wright nebulizer, was introduced in the 1950s. Plastic molding enabled improved precision of the venturi orifice and produced much finer sprays capable of reaching the deep lung and resulted in performance similar to modern nebulizers.^(28)^ Thus, in the century from 1850 to 1950, nebulizer technology had advanced from infancy to devices capable of delivering highly respirable aerosols for effective treatment of lung diseases.

### Early DPIs

**FIG. 6.**
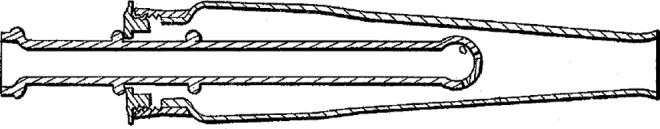
A drawing of the first DPI invented by Ira Warren in 1852.

The first known DPI was invented by Boston physician Ira Warren in 1852^(37)^ (Fig. 6 below). The glass inhaler consists of an inner tube pierced with fine holes and in which the powdered medicine is placed. The inner tube was enclosed in an outer tube with a mouthpiece (on the right side of Fig. 6). The inner tube was twirled by hand as the patient inhaled, causing the powder to be aerosolized through the holes in the inner tube and delivered to the patient through the mouthpiece. In the patent, Warren states that the powder inhaler is designed for the purpose of inhaling medicine into the throat and lungs and, at the same time, to prevent any of the said medicine from lodging in the mouth. The inhaler sold for $1 along with vials of powder to be inhaled, which could be purchased for $0.50 per vial. Powders available for purchase included silver nitrate, copper sulfate, and mercury nitrate (advertisement in The Boston Medical and Surgical Journal, November 3, 1852).

Perhaps the most interesting early DPI device was the Carbolic Smoke Ball, which was invented by Frederick Roe in 1889.^(3)^ The device consisted of an ∼5 cm hollow ball made of India rubber and a 1.5-cm-long nozzle made of vulcanite with a fine gauze placed ∼0.5 cm from the end of the nozzle.^(38)^ Inside the ball was a powder composition consisting of glycyrrhiza, hellebore, and carbolic acid.^(3,38)^ When the patient squeezed the rubber ball, the powder was aerosolized and deagglomerated by shear forces as it passed through the sieve in a manner similar to the Rotahaler DPI device introduced approximately a century later. The patient was instructed to inhale the smoke-like aerosol that was formed.^(38)^

The device was marketed by the London-based Carbolic Smoke Ball Company, which certainly did not lack boldness in their marketing department! Advertisements for the device provided a long list of diseases that they claimed could be cured with the Carbolic Smoke Ball and even provide the time required to achieve the cure—“Carbolic Smoke Ball positively will cure: Coughs (cured in 1 week), cold in the head (cured in 12 hours), cold on the chest (cured in 12 hours), catarrh (cured in 1–3 months), asthma (relieved in 10 minutes), bronchitis (cured in every case), hoarseness (cured in 12 hours), loss of voice (fully restored), sore throat (cured in 12 hours), throat deafness (cured in 1–3 months), snoring (cured in 1 week), sore eyes (cured in 2 weeks), influenza (cured in 24 hours), hay fever (cured in every case), headache (cured in 20 minutes), croup (cured in 5 minutes), whooping cough (relieved first application), and neuralgia (cured in 20 minutes)” (from an advertisement in Vanity Fair on January 23, 1892). The advertisement offered a £100 reward to anyone who contracted influenza despite using a Carbolic Smoke Ball.

Eventually, the cavalier advertisements caught up with the Carbolic Smoke Ball Company when Miss E.C. Carlill of London fell ill with influenza in spite of having judiciously taken her Carbolic Smoke Ball in January of 1892. The defendant argued that their claims constituted puffery rather than a binding offer since the plaintiff had not communicated acceptance of the offer.^(39)^ That line of defense was obliterated by the judge who ruled that “the defendants must perform their promise and, if they have been so unwary as to expose themselves to a great many actions, so much the worse for them.”^(39)^

Another notable DPI during this era was Alfred Newton's patented DPI for delivery of finely pulverized powders.^(3,40)^ While Newton's choice of potassium chlorate powders for treating respiratory diseases was not ideal (since this is now known to be a lung irritant), Newton recognized the importance of minimizing powder exposure to moisture to achieve effective delivery of the powder to the lung.^(3)^

**FIG. 7.**
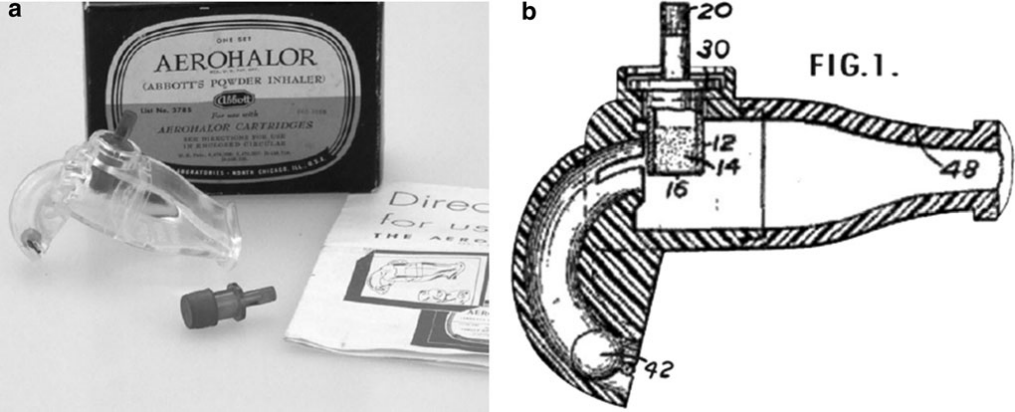
A picture **(a)** and schematic drawing **(b)** of Abbott's Aerohalor. Images courtesy of Mark Sanders.

Perhaps the first truly commercially successful DPI was the Aerohalor DPI that was developed by Mack Fields of Abbott Laboratories.^(41)^ This was used to deliver the beta agonist isoprenaline sulfate under the brand name Norisodrine^®(27)^ and was also used to deliver penicillin for treatment of respiratory infection.^(42)^ The device utilized a steel ball that moved when the patient inhaled and tapped a cartridge that contained the drug to aerosolize the powder (Fig. 7). The device contained a mouthpiece or nasal adapter depending on the desired route of delivery. The device was a breakthrough in terms of commercial viability of a DPI device in spite of the fact that it was relatively inefficient in terms of dispersing the powder into a respirable aerosol.^(27)^

### Precursors to the modern era of pharmaceutical aerosols

In addition to advancements in devices for delivery of therapeutic aerosols, other advancements in the first half of the 20th century helped set the stage for the onset of the modern era of pharmaceutical aerosol delivery. Three particularly significant advancements were the discovery of new drugs for treatment of respiratory diseases, the passing of the 1906 Food and Drug Act, and the proliferation of clinical trials for evaluating the effectiveness of these drugs.

Two particularly important classes of drugs first delivered through aerosol during this period were beta agonists and corticosteroids—the two most widely used classes of drugs currently prescribed for the treatment of asthma. In 1900, Solomon Solis-Cohen demonstrated that tablets containing a crude extract of the adrenal gland that likely contained both the beta agonist epinephrine (also known as adrenaline) and the corticosteroid cortisone induced bronchodilation.^(43)^ In 1910, inhaled epinephrine was shown by Barger and Dale to be therapeutically effective for the treatment of asthma.^(9)^ Inhaled epinephrine delivered by nebulizer rapidly became a prominent therapy for treatment of asthma. The beta agonist isoprenaline (isoproterenol) delivered using the Aerohalor DPI was first commercialized in 1948.

These nonselective beta-adrenergic agonists were effective bronchodilators, but had significant side effects. The first published study on the use of inhaled corticosteroids is a study by Reeder and Mackay in 1950 that demonstrated that nebulized cortisone was effective at bringing about the remission of symptoms of bacterial pneumonia.^(44)^ In 1951, Gelfand demonstrated the effectiveness of inhaled cortisone for the treatment of asthma.^(45)^

The signing of the Food and Drug Act of 1906 reshaped the development and testing of therapeutic aerosols. As a result, there was a great increase in the execution and publication of controlled clinical trials to evaluate the effectiveness of various therapeutic aerosols. Excellent summaries of some of the important published trials during this era can be found elsewhere.^(3,19,28,42)^

Thus, by the mid-1950s, the aerosol delivery of beta agonists, corticosteroids, and anticholinergic (i.e., atropine contained in stramonium) drugs had all been demonstrated to be effective for the treatment of respiratory diseases. Convenient delivery by a DPI or squeeze bulb glass nebulizers had been demonstrated, although with limited ability to generate respirable aerosols. More efficient nebulizer systems had been developed capable of delivering drugs to the deep lung, but these were not portable and convenient. The stage was set for the development of pharmaceutical inhalers that were both convenient and able to deliver drugs to the deep lung.

## The Delivery of Therapeutic Aerosols from 1956 to 1986

The period from 1956 to 1986 marked the modernization of the delivery of therapeutic aerosols and the introduction of pharmaceutical aerosol devices that are widely used today. The introduction of the MDI in 1956 was a major breakthrough in the treatment of respiratory diseases, particularly asthma. The MDI was the first inhaler device that achieved effective lung delivery in a truly convenient and portable device and rapidly became the dominant delivery system for treatment of asthma. Add-on devices were introduced to improve the efficiency of MDIs and to overcome challenges associated with patient coordination. While MDIs dominated the market during this period, two important new DPI devices were commercialized during this period and important insight was gained into the development of suitable DPI formulations. In addition, the introduction of albuterol was a breakthrough in asthma therapy. Other notable drugs introduced for inhalation during this period include cromolyn sodium (also known as sodium cromoglycate) and the steroid, beclomethasone dipropionate.

### The breakthrough of the MDI

In April of 1955, a young girl named Susie Maison was displeased with her squeeze bulb nebulizer that she used to treat her asthma and asked her father, George Maison, MD, President of Riker Laboratories, the question, “Daddy, why can't they put my asthma medicine in a spray-can like they do hair spray?” A serendipitous convergence of circumstances resulted in this simple question leading to the development of an important new therapy, the MDI.^(46)^ Remarkably, this new therapy has improved the quality of life of hundreds of millions of people around the world and has saved countless lives.

**FIG. 8.**
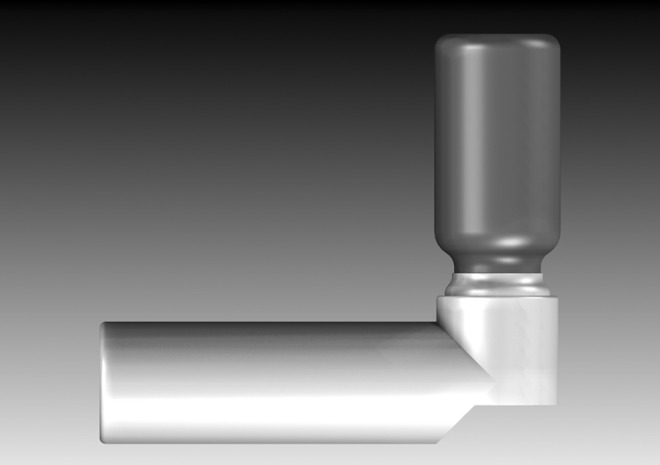
A drawing of the first MDI, Medihaler Iso, which consisted of a plastic-coated glass vial crimped to a 50 mcl metering valve, the formulation, and the plastic mouthpiece adapter.

Within 2 months of Susie's question, Riker Laboratories began clinical testing on MDI formulations of isoproterenol and epinephrine using solution formulations (developed by Irving Porush of Riker) containing a mixture of Freon 12™ and Freon 114™ with 35% w/w ethanol. Figure 8 shows a drawing of the first MDI. On January 12, 1956, New Drug Applications were filed for Medihaler Epi (epinephrine) and Medihaler Iso (isoproterenol). With the approval of Medihaler Epi and Medihaler Iso on March 9, 1956, both products were launched before the end of the month!

Acceptance of the new delivery system by physicians and patients was rapid, and in December of 1956, the first clinical evaluation of the MDI was published. Freedman evaluated 42 asthmatics and reported that 30 of the patients obtained good to excellent relief of symptoms and five others obtained fair relief.^(47)^ Freedman remarked that this response to Medihaler therapy is remarkable in view of the fact that most of the patients had failed to respond satisfactorily to other commonly employed therapeutic measures.

Encouraged by the successful launches of Medihaler Iso and Epi, Riker began to apply their MDI platform to treat other indications. Medihaler Nitro (octyl nitrate) was launched in late 1956 for the treatment of angina. While therapeutically effective (it aborted angina attacks within about 20 seconds of inhaling a dose), it was withdrawn early in 1958 since most patients were willing to endure several minutes of pain before relief to discretely slip a nitroglycerine tablet under their tongue rather than use an inhaler.^(46)^ The first nasal spray MDI, Medihaler Phen, was commercialized in the spring of 1957. Medihaler Phen was a triple-combination therapy that delivered phenylephrine, neomycin, and hydrocortisone and was the first MDI formulation in which the drugs were formulated as suspensions since solution formulations with 35% ethanol were irritating to the nose. The clinical pathway for approval of this triple-combination formulation most certainly was simpler than the regulatory requirements for clinical evaluation of triple-combinations currently under development!

Medihaler Phen was not a commercial success since it sold on prescription for $3.50 and had to compete with an effective over-the-counter 69-cent squeeze bottle. Nevertheless, the suspension formulations would prove to be an important advance in MDI formulation technology. Suspension formulations were capable of delivering much more drug to the lung and allowed for the formulation of many other drugs. Medihaler Ergotamine was approved for the treatment of migraine headache in 1959. Medihaler Duo, the first combination MDI therapy for treatment of asthma, delivered isoproterenol hydrochloride and phenylephrine bitartrate and was approved in 1962.

During the late fifties and sixties, a wide variety of drugs were evaluated in MDIs, but most never made it to the marketplace. Insulin MDI formulations were demonstrated to elicit a hypoglycemic response, but development was not continued as the delivery at that time was considered too variable for safe use in humans.^(46)^ Among other MDI formulations evaluated were formulations of BCG (Bacillus Calmette–Guérin) vaccine for treatment of TB, live measles vaccine, Methylphenidate for treatment of attention deficit disorder, and Triiodothyronine as a hangover cure. Riker was even asked to formulate an MDI of sulfur dioxide for an oncologist who wished to use it to cause patients to cough violently enough to bring up cells as a lung cancer diagnostic test!

One of the primary limitations of the MDI is the challenge that some patients have coordinating their actuation of the device with their inhalation maneuver. In his initial clinical evaluation on the MDI, Freedman indicated that the primary cause of poor response with Medihaler was the failure of physicians to stress to the patient the importance of synchronization of inspiration with the administration of the dose.^(47)^

**FIG. 9.**
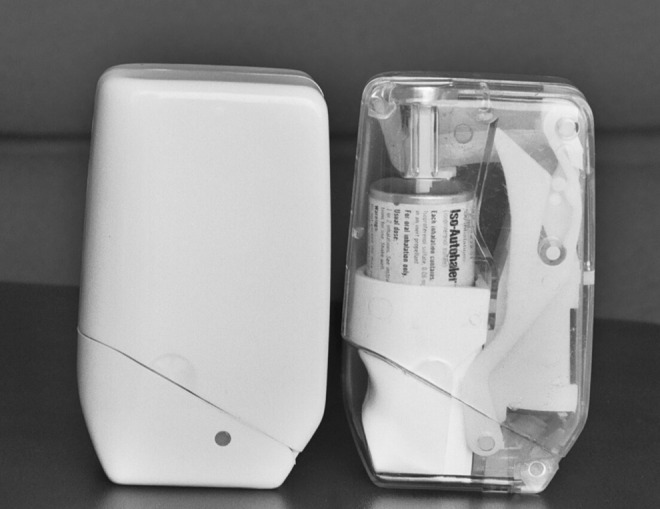
A photograph of the first commercial breath actuated MDI, the Autohaler, along with a transparent demonstrator device.

As the awareness grew that some patients had difficulty synchronizing the release of a dose from an MDI with inhalation, the desirability of a breath-actuated MDI became apparent. The first breath-actuated MDI, the Autohaler, was commercialized by Riker in 1970 as the Duohaler (isoproterenol hydrochloride and phenylephrine bitartrate) and was followed shortly by the Iso-Autohaler (isoproterenol). The Autohaler was a rugged pocket-sized device that patients found convenient and simple to use, but was only marginally commercially successful.^(46)^ Figure 9 shows the first Autohaler device.

Early MDI formulations were relatively inefficient and resulted in a significant portion of the delivered aerosol consisting of large droplets that deposited in the mouth and throat of the patient. In 1958, Franklin et al. demonstrated that deposition in the mouth and throat could be significantly reduced by using add-on devices—in their case, a 1” diameter plastic tube that was 14” long.^(47a)^ Physicians trained patients to use a variety of homemade add-on devices, including empty vinegar bottles, toilet paper tubes, plastic cups, and paper bags.^(48)^ An early spacer device that was widely used was the Breathancer^®^ tube spacer introduced by Ciba-Geigy in the late 1970s.^(49)^ Beginning in the late 1970s, valved holding chambers were developed to help contain the aerosol before inhalation for patients with poor coordination or who are to be administered doses during tidal breathing.^(50)^ In addition to reducing mouth and throat deposition,^(51)^ large volume add-on devices have been shown to increase the total drug deposition in the small airways.^(52)^ A wide variety of spacer and holding chambers are commercially available today.^(48)^ A detailed summary of the interesting history of spacers and holding chambers can be found elsewhere.^(50)^

In the 1970s and 1980s, MDIs continued to grow in popularity with the introduction of several important MDI products. In 1972, Allen & Hanburys commercialized the first beclomethasone dipropionate MDI (Becotide^®^) in Europe. Later, in 1982, beclomethasone dipropionate MDIs were marketed in the United States by Schering Corporation (Beclovent) and Glaxo Wellcome (Vanceril). Boehringer Ingelheim introduced Alupent (metaproterenol sulfate) in 1973. Two albuterol MDIs were introduced in 1981, Proventil by Schering Corporation and Ventolin by Glaxo Wellcome. Aerobid (flunisolide) was introduced by Forest in 1984. Atrovent (ipratropium bromide) was introduced by Boehringer Ingelheim in 1986. Table 1 shows a list of MDI products approved by the U.S. FDA since 1956 that use chlorofluorocarbon (CFC) propellants.

**Table 1.** A Partial List of CFC Metered Dose Inhaler Products Approved by the U.S. FDA

*Year, product name*	*Drug(s)*	*Company*
1956, Medihaler Epi	Epinephrine	Riker
1956, Medihaler Iso	Isoproterenol	Riker
1956, Medihaler Nitro	Octyl nitrate	Riker
1957, Medihaler Phen	Phenylephrine/neomycin/hydrocortisone	Riker
1959, Medihaler Ergotamine	Ergotamine	Riker
1962, Medihaler Duo	Isoproterenol hydrochloride/phenylephrine bitartrate	Riker
1970, Duohaler	Isoproterenol hydrochloride/phenylephrine bitartrate	Riker
1970, Iso-Autohaler	Isoproterenol	Riker
1973, Alupent	Metaproterenol sulfate	Boehringer Ingelheim
1981, Proventil	Albuterol	Schering
1981, Ventolin	Albuterol	Glaxo Wellcome
1982, Beclovent	Beclomethasone dipropionate	Schering
1982, Vanceril	Beclomethasone dipropionate	Glaxo Wellcome
1982, Azmacort	Triamcinolone acetonide	Rhône-Poulenc
1984, Aerobid	Flunisolide	Roche
1985, Intal	Cromolyn sodium	Fisons
1986, Atrovent	Ipratropium bromide	Boehringer Ingelheim
1986, Maxair	Pirbuterol acetate	3 M
1992, Maxair Autohaler	Pirbuterol acetate	3 M
1995, Generic albuterol	Albuterol	IVAX
1996, Generic albuterol	Albuterol	Pliva
1996, Combivent	Albuterol/ipratropium	Boehringer Ingelheim
1996, Generic albuterol	Albuterol	Armstrong
1997, Generic albuterol	Albuterol	GenPharm

### The breakthrough of albuterol

A second major breakthrough in the treatment of asthma during this period was the introduction of albuterol (also known as salbutamol). Albuterol was important since it was the first selective β_2_ agonist developed. Research in the early 1960s identified that beta adrenoceptors had two subtypes—β_1_ receptors, which are primarily found in the heart, and β_2_ receptors, which are primarily found in the lungs.^(53)^ Previous beta agonists, such as isoprenaline, effectively achieved bronchodilation, but caused significant side effects such as tachycardia due to their lack of β_2_ selectivity. Researchers at Allen & Hanburys created albuterol, an analog of isoprenaline, which had a potency 500 times greater for β_2_ compared with β_1_ receptors.^(54)^ Ventolin, an MDI delivering albuterol, was commercialized in 1968 and was followed by albuterol nebulizer solutions and DPIs.^(3)^ Albuterol remains a critical rescue therapy used by millions of asthmatics.

### Advances in DPI and nebulizer technologies

**FIG. 10.**
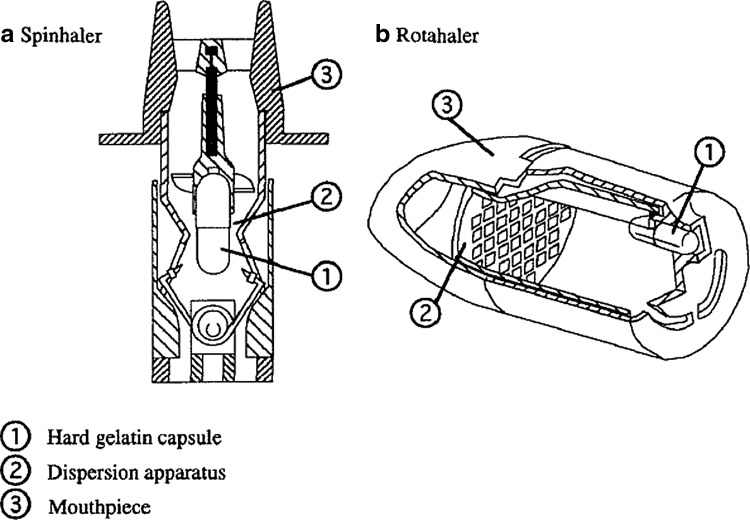
Schematic drawings of two early DPI devices. Images courtesy of Andy Clark.

While somewhat overshadowed due to the emergence of the MDI, there were advances in DPI and nebulizer technologies during the period from 1956 to 1986. The Intal Spinhaler delivering cromolyn sodium (sodium cromoglycate) was introduced by Fisons in the United Kingdom in 1967 and in the United States in 1970. The Spinhaler DPI delivered powder from a gelatin capsule that is inserted into a cavity connected to an impeller (Fig. 10a). The capsule is punctured by a piercing apparatus that is actuated by a movement of the device body by the patient before dosing. When the patient inhales, the rotation of the impeller causes significant movement of the powder in the capsule that transfers the powder out of the capsule and into the airstream where interaction with the rotating impeller further disperses the powder into fine particles suitable for inhalation.^(55)^ Bell demonstrated the need to include a larger carrier particle (in particular, lactose with a median particle size between 30 and 60 μm) to achieve effective delivery of cromolyn sodium using the Spinhaler. This formulation approach remains a critical aspect of DPI formulation development today.

The Rotahaler DPI was introduced by Allen & Hanburys in 1977 for the delivery of albuterol.^(27)^ The Rotahaler uses a rotation of the two parts of the inhaler to separate two halves of a capsule containing the drug (Fig. 10b). Some of the contents of these capsules empty when the patient inhales and are dispersed through a simple plastic mesh molded in one of the halves of the inhaler. Both of these devices were relatively inefficient at delivering drug to the lung.

Nebulizers remained an important part of mainstream inhalation therapy between 1956 and 1986. With improved injection molding capabilities and oil-free compressed air pumps available, a broad range of disposable nebulizer options were commercialized during this period. In addition, the output characteristics of jet nebulizers were studied and improved through optimization of the air jet, capillary tube, and baffle design.^(56–58)^ A notable innovation during this period was the introduction by Robert Lang in 1962 of a nebulizer that utilized a piezoelectric crystal to atomize the solution.^(59)^ Lang showed that it was possible to generate high-frequency ultrasonic vibrations that atomized the liquid into finer aerosols than could typically be achieved using jet nebulizers. This approach had the advantage of being smaller and easier to use, as well as being able to more rapidly deliver aerosols capable of reaching the deep lung. However, the uptake of this technology was limited by the marginal reliability of initial piezoelectric nebulizers and the strain on the piezoelectric crystal caused by the changing load as the fluid is delivered.^(27)^

## The Delivery of Therapeutic Aerosols from 1987 to Present

The period from 1987 to the present has been a period of unprecedented innovation and growth in the delivery of therapeutic aerosols. Annual sales of pharmaceutical inhalers have increased from $7 billion^(60)^ in 1987 to $36 billion in 2014^(32)^ and the number of inhaled doses sold to patients in 2014 exceeded 90 billion.^(32)^ The number of pharmaceutical products available to deliver drug to the respiratory tract has grown dramatically. The signing of the Montreal Protocol^(61)^ in September of 1987 dramatically changed the pharmaceutical aerosol industry and led to a surge of development and innovation of inhaler products that eventually resulted in hydrofluoroalkane (HFA) MDIs and a large increase in the number and types of DPIs available, as well as the development of advanced nebulizer systems and other inhalation devices.

Several important changes in the market dynamics transformed the landscape of the pharmaceutical aerosol industry during this period. The surge in innovation was complicated by a dramatic increase in the regulatory burden required to gain market approval of new inhaler devices. The development of improved drugs and delivery systems, along with aggressive marketing, brought about the rise of blockbuster therapies—particularly combination therapies containing a long-acting beta agonist (LABA) and a corticosteroid. Last, the era since 1987 was marked by the monumental efforts to develop insulin inhalers for noninvasive treatment of diabetes.

### The Montreal protocol and the explosion of innovation

By the 1980s, MDIs had become the preferred delivery system for the delivery of therapeutic aerosols to the lung. The extremely inert nature of CFC propellants used in MDIs (and in many other industrial applications at the time) enabled these molecules to diffuse over time into the upper stratosphere. An article published in 1974 by Molina and Rowland^(62)^ demonstrated that CFC propellants break down and release chlorine radicals when exposed to sunlight in the upper stratosphere. These chlorine radicals were shown to have the ability to break down very large numbers of ozone molecules. In the mid-1980s, evidence mounted that stratospheric ozone levels were decreasing at an alarming level and that CFCs were contributing significantly to this depletion.

In 1987, the Montreal Protocol was signed and called for the elimination of CFC propellants with a date of January 1996 eventually agreed upon. Although orally inhaled MDI products were exempt from this ban until medically acceptable alternatives were available, the impact on the pharmaceutical aerosol industry of the Montreal Protocol was dramatic. The impending elimination of CFC propellants led to a race in the industry to develop alternative inhaler devices to replace CFC-based MDIs.

**FIG. 11.**
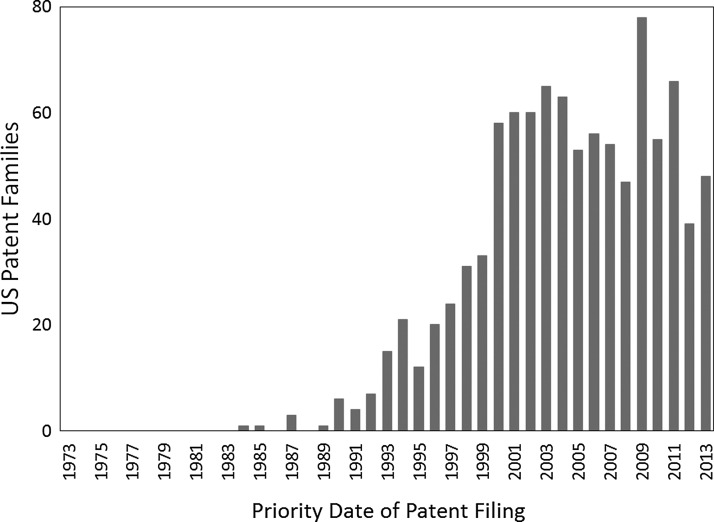
The number of U.S. patent families containing the phrase “metered dose inhaler or dry powder inhaler” in the title, claims, or abstract plotted against the year of the priority filing.

Figure 11 shows the number of U.S. patent families with the term “metered dose inhaler” or “dry powder inhaler” in the title, abstract, or claims over the years from 1973 to 2013 as a function of their priority filing date. Many other inhaler-related patents are not included in this list because they do not use either of these terms or they were filed in other countries. Whereas patent activity in the field of pharmaceutical inhalers had been relatively stagnant in the 1970s and early 1980s, there was a rapid increase in patent activity starting in about 1990. Given that there is usually a lag between investment in innovation and the filing of a patent application, Figure 11 illustrates that the signing of the Montreal Protocol was quickly followed by a surge of innovation in therapeutic aerosol delivery technologies. Some of the key areas of innovation are discussed in the subsequent sections.

### The development of HFA MDIs

Pharmaceutical companies began to search for new propellants to replace CFCs for use in MDIs. Atkins^(63)^ indicates that thousands of different compounds were evaluated. Nonchlorinated HFAs were identified as promising candidates and HFA-134 and HFA-227 were quickly identified as top candidates to replace CFCs. Several industry consortia were established to collaboratively generate the data needed to demonstrate the safety of these new propellants to the level expected based on heightened regulatory requirements.^(63)^

While there was great collaboration in generating toxicology data on HFA propellants, there was great competition among the companies to obtain patent positions. An excellent description of the patent landscape related to HFA MDIs can be found elsewhere.^(64)^ The race to develop patent positions was influenced by, among other things, the ability to obtain HFA propellant samples from propellant manufacturers. Thiel^(46)^ describes the importance of obtaining a sample of HFA-134a in early 1988 and how this sample helped identify the utility of ethanol as a cosolvent to solubilize traditional MDI surfactants, such as oleic acid. This early discovery helped establish the 3 M Riker patent portfolio. The first HFA MDI patent application was filed by 3 M Riker in 1988 and described MDIs free of CFC propellant, using HFA-134a, a surface active agent, and a polar cosolvent.^(65)^

**FIG. 12.**
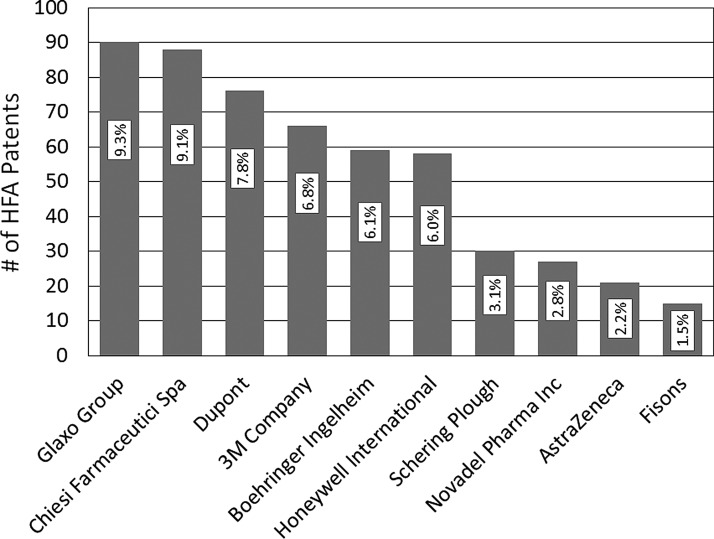
The number of published HFA MDI patents for the 10 companies with the largest patent portfolios (with the percentage of all HFA MDI patents). Adapted from Rogueda et al.^(64)^

The first two patent applications describing MDIs using HFA-227 were filed in 1990 by Solvay Fluorochemicals and Boehringer Ingelheim.^(66,67)^ Figure 12 shows the patent portfolios for the 10 companies with the largest HFA MDI patent portfolios. Rogueda et al. concluded that the field is controlled by early players, leaving little space for newcomers.^(64)^ Indeed, nearly half of the patents are held by four pharmaceutical companies (GSK, Chiesi, 3 M, and Boehringer Ingelheim) and two propellant manufacturers (Dupont and Honeywell).

The transition from CFC to HFA propellants was not straightforward and involved significant investment in research and development.^(60)^ The renewed investment required to transition to HFA propellants resulted in improvements to the MDI device components as well. Elastomeric components of the valve used in CFC MDIs were not compatible with the HFA propellants. The new elastomers developed, such as EPDM (ethylene propylene diene monomer), were cleaner and resulted in significantly reduced leachable levels.^(68)^ The change in elastomers brought about redesigned MDI valves, which resulted in significantly improved end-of-unit dosing consistency.^(69)^ Coated canisters were developed to minimize drug deposition that is prevalent for ethanol-free HFA suspension formulations and to reduce chemical degradation for solution MDI formulations.^(68,70,71)^ High-efficiency actuator devices were developed to manipulate or contain the plume in such a way as to reduce oropharyngeal deposition while increasing lung deposition.^(68,72,73)^

The first HFA MDI product to reach the market was an albuterol sulfate suspension in HFA-134a developed by 3 M. The product was approved in the United Kingdom in 1994 and commercialized in 1995 as Airomir. The product was approved and launched in the United States in 1996 as Proventil HFA. The product was designed to provide similar drug delivery to the CFC product (which was also a suspension), although some patients noticed a slight difference in taste.^(46)^

The first HFA corticosteroid to reach the market was Qvar, an HFA-134a formulation of beclomethasone dipropionate commercialized by 3 M in 2000. Due to the physical and chemical properties of beclomethasone dipropionate, it was not possible to develop a stable suspension formulation, so the product was formulated using ethanol as a cosolvent to solubilize the drug. The solution formulation provided an extrafine aerosol with a much smaller mass median aerodynamic diameter than the CFC beclomethasone suspension Beclovent (1.1 vs. 3.5 μm, respectively).^(60)^ The extrafine nature of Qvar was shown to provide greatly enhanced deep lung deposition^(74)^ and has subsequently been shown to provide improved therapeutic outcomes.^(75–77)^ Other HFA steroid solution MDI products have been approved, Alvesco (ciclesonide) by Nycomed and Aerospan HFA (flunisolide) by Forest, and have shown similar therapeutic benefits.^(76,77)^

In 2001, GSK received approval from the FDA for Ventolin HFA, which was a suspension of albuterol sulfate free of surfactant and ethanol. The first HFA-227 MDI to receive approval from the FDA was AstraZeneca's Symbicort (budesonide and formoterol fumarate), which was approved in 2006. A significant number of other HFA MDI products have received FDA approval (Table 2) and many more HFA MDI products are approved in other regions of the world. MDIs remain the most widely used delivery system for treating respiratory diseases with more than 75 billion doses sold in 2014.^(32)^

**Table 2.** A Partial List of Hydrofluoroalkane Metered Dose Inhaler Products Approved by the U.S. FDA

*Year, product name*	*Drug(s)*	*Company*
1996, Proventil HFA	Albuterol sulfate	3 M
2000, QVAR	Beclomethasone dipropionate	3 M
2001, Ventolin HFA	Albuterol sulfate	GlaxoSmithKline
2004, Proair HFA	Albuterol sulfate	Ivax
2004, Atrovent HFA	Ipratropium bromide	Boehringer Ingelheim
2005, Xopenex HFA	Levalbuterol tartrate	Sepracor
2006, Aerospan	Flunisolide	Forest
2006, Advair HFA	Fluticasone propionate/salmeterol	GlaxoSmithKline
2006, Flovent HFA	Fluticasone propionate	GlaxoSmithKline
2006, Symbicort	Budesonide/formoterol	Astra Zeneca
2008, Alvesco	Ciclesonide	Nycomed/Sunovion
2010, Dulera	Mometasone/formoterol fumarate	Merck
2014, Asmanex HFA	Mometasone furoate	Merck

HFA, hydrofluoroalkane.

In 2004, GSK launched Seretide™ Evohaler™ (salmeterol and fluticasone propionate) in Europe. This was the first MDI with an integrated dose counter incorporated into the device to help patients know when their device has exceeded or is near the last available dose. The FDA published a Guidance for Industry in 2003^(78)^ requiring all new MDI products to have dose counters or dose indicators.

The Montreal Protocol's Essential Use exemption for HFA MDIs was intended to ensure continuity of supply of critical asthma medicines until medically acceptable alternatives were available. As HFA MDIs began to reach the market, countries began implementing phaseout schedules of CFC products. Albuterol CFC MDIs were phased out in the United States at the end of 2008, all CFC MDIs were phased out in Europe by 2010, and the last CFC MDI (Primatene Mist) on the U.S. market was phased out at the end of 2011.^(79,80)^

If we assume that the ∼400 million HFA MDIs sold in 2014^(32)^ replaced CFC MDIs (10 g fill weight of 30% CFC-11 and 70% CFC-12), the result is a reduction of nearly 2600 metric tons of chlorine released into the atmosphere. However, the environmental benefit of the CFC MDI phaseout came with the cost of eliminating access to cheaper generic inhalers for many people.^(60)^ The Montreal Protocol does allow developing countries (Article 5) to continue to use CFC MDIs for a longer duration to ensure affordable inhalers are available in these markets. As a result, at the time of the writing of this article, there are still limited amounts of CFC MDIs on the market in some countries.

### The emergence of DPIs

In 1987, the DPIs available on the market were inefficient single-dose reloadable inhalers and accounted for a relatively small fraction of the inhaler market. Since 1987, the number of DPIs available has dramatically increased. Table 3 shows a list of DPIs that have been approved by the FDA. Many other DPIs are in development or are available in other markets.

**Table 3.** A Partial List of Dry Powder Inhalers That Have Been Approved by the U.S. FDA

*Year, product name*	*Drug(s)*	*Company*
1940s, Aerohalor	Isoproterenol sulfate	Abbott
1970, Spinhaler	Cromolyn sodium	Fisons
1988, Ventolin Rotacaps	Albuterol	Glaxo
1997, Flovent Diskus	Fluticasone	Glaxo Wellcome
1997, Serevent Diskus	Salmeterol	Glaxo Wellcome
1997, Pulmicort Turbuhaler	Budesonide	Astra Zeneca
1999, Relenza Diskhaler	Zanamivir	Glaxo Wellcome
2000, Advair Diskus	Fluticasone/salmeterol	GlaxoSmithKline
2001, Foradil Aerolizer	Formoterol fumarate	Schering Plough/Novartis
2004, Spiriva Handihaler	Tiotropium bromide	Boehringer Ingelheim
2005, Asmanex Twisthaler	Mometasone furoate	Schering Plough
2006, Exubera	Recombinant human insulin	Nektar/Pfizer
2006, Pulmicort Flexhaler	Budesonide	Astra Zeneca
2006, Foradil Certihaler	Formoterol fumarate	Schering Plough/Novartis
2010, Aridol	Mannitol	Pharmaxis
2011, Arcapta Neohaler	Indacaterol maleate	Novartis
2013, Tobi Podhaler	Tobramycin inhalation powder	Novartis
2013, Breo Ellipta	Fluticasone furoate/vilanterol	GlaxoSmithKline
2014, Incruise Ellipta	Umeclidinium	GlaxoSmithKline
2014, Afrezza	Human recombinant insulin	MannKind

There has been significant enhancement in the functionality and convenience of the device used in many DPI products developed since 1987. Early DPI devices, such as the Spinhaler and Rotahaler, had each dose stored in an individual capsule that the patient needed to have present and load at the time of dosing. Many DPIs commercialized since 1987 are inherently multidose and require reduced patient manipulation and handling before dosing. The first multidose DPIs, the Serevent Diskhaler™ and Pulmicort Turbuhaler™/Turbohaler™, were commercialized in Europe in 1988 by Glaxo and Astra, respectively. The Diskhaler™ used disks with 4 or 8 individual doses of a lactose/drug blend with each dose isolated in a separate foil blister. When the patient opened the mouthpiece cover of the device before each dose, a mechanism pierced the blister, thus making the powder available for inhalation.

**FIG. 13.**
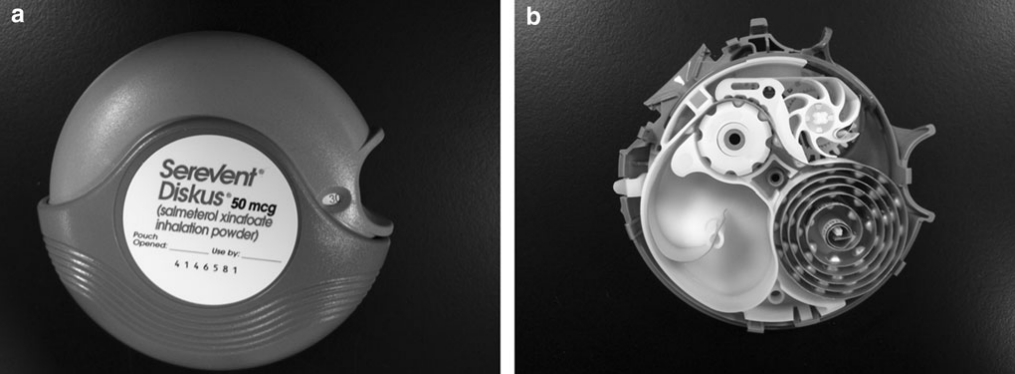
The Diskus DPI as provided to the patient **(a)** and after disassembly to show the coil dosing strip containing the 60 individual foil blisters [bottom right portion of the image **(b)]**.

The Turbohaler™ contained a reservoir of pure drug powder sufficient to deliver up to 200 doses. Before each dose, the patient twisted the base of the inhaler to rotate a disk that had small cylinders that were filled with powder from the reservoir and then emptied as the patient inhaled to deliver the drug. The Diskus™ DPI, introduced by Glaxo Wellcome in the mid-1990s, contained a blister strip with 60 individually sealed doses of a lactose/drug blend (Fig. 13). To operate the device, the patient rotates the mouthpiece cover and then slides a lever that advances the next blister to the dosing position and peels the two sides of the foil blister, thus making the powder available for inhalation.^(81)^ The improved usability of modern DPI devices has resulted in DPIs playing an increasingly important role in the treatment of respiratory diseases.^(82)^

The cohesive nature of inhalation powders makes it necessary to impart significant energy to the powder to efficiently disperse and deagglomerate the powder such that the drug is capable of reaching the lung. As DPI device technologies multiplied, so did the different approaches to deagglomerate the powder. Most approaches utilize the energy of the patient's inhalation flow (passive energy) to deagglomerate the powder.^(83)^ In addition, a few DPI devices were developed utilizing active energy sources to deagglomerate the powder, thus enabling efficient delivery even for patients with compromised inhalation flowrate. The first active DPI to reach the market was the Exubera DPI, which utilized compressed air to deagglomerate the powder, and was launched in 2006. Numerous other active DPI technologies have been developed or are still in development. The Spiros^®^ DPI developed by Dura Pharmaceuticals utilized a battery-powered impeller to efficiently deagglomerate the powder,^(84)^ but failed to achieve regulatory approval and development was discontinued. MicroDose Technologies and Oriel Therapeutics both developed DPIs that utilize piezoelectric vibrational energy to efficiently deagglomerate the powder.

The advancements in DPI device technology since 1987 were matched by improvements in DPI formulations. To be suitable for use in a DPI, a powder must have flow properties that enable consistent dose metering by the device or in the manufacturing facility.^(85)^ In addition, the powder must be capable of deagglomerating such that the drug particles are delivered to the patient in a respirable form. Formulations before 1987 were marginally acceptable in meeting this criterion. Increased understanding of the interparticulate interaction of inhalation powders led to numerous technical advances in DPI formulation.

In 1996, Staniforth^(86)^ investigated ordered blend DPI formulations in which the drug particles adhered to the surface of much larger excipient particles (typically lactose monohydrate). He concluded that DPI delivery efficiency was limited by the fact that a significant portion of the micronized drug particles permanently adhered to high energy sites on coarse lactose particles and thus were not capable of reaching the deep lung. The delivery efficiency from ordered blend formulations containing drug and coarse lactose was found to significantly increase when fine lactose was included in the formulation.^(87–90)^ These ternary formulations are now a key DPI formulation approach.

An alternate formulation approach to using fine lactose that gained prominence in the late 1990s is to use a force control agent to reduce the propensity of micronized drug particles to permanently adhere to high energy sites on the lactose carrier particles.^(86)^ Vectura's Powderhale and Skyepharma's SkyeProtect™ technologies, utilizing magnesium stearate, are examples of this approach and have been shown to improve the flow of ordered blends and enhance the release of micronized drug from the carrier particles.^(91,92)^

**FIG. 14.**
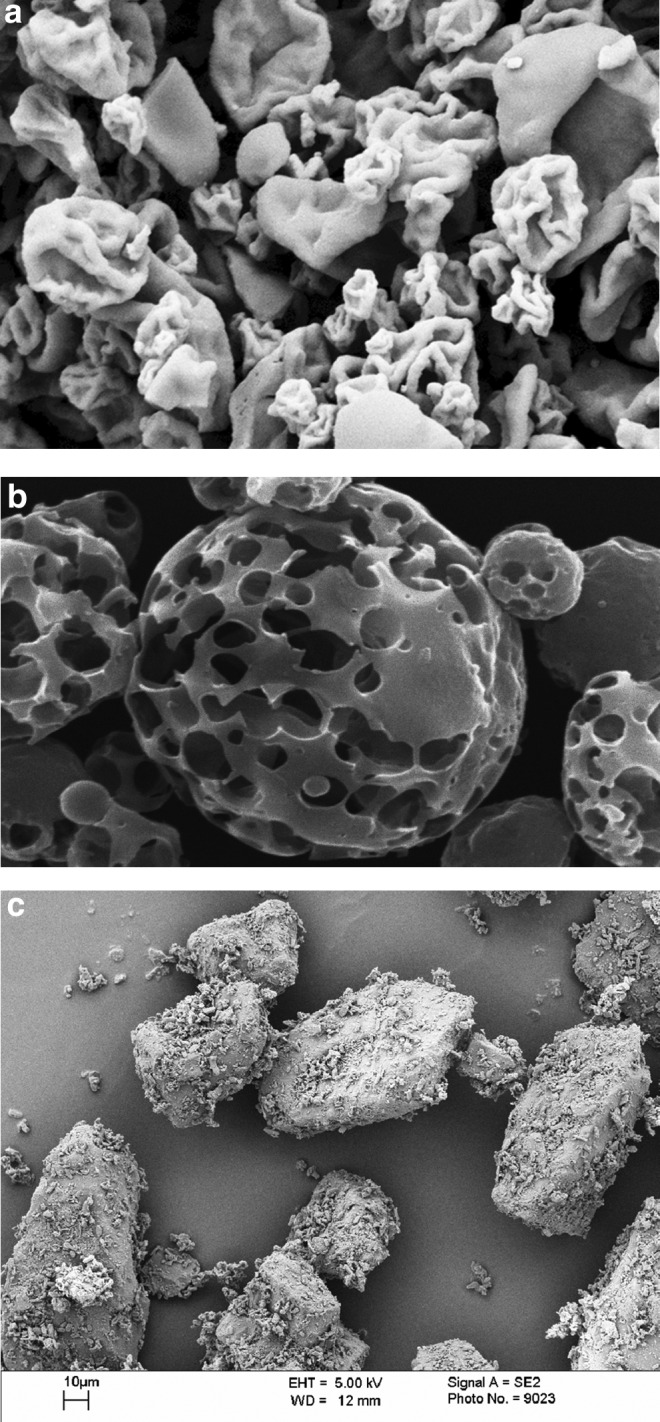
Powders harvested from commercial DPIs. **(a)** Spray-dried recombinant human insulin from Exubera. **(b)** Pulmospheres™ from Tobi^®^ Podhaler™. **(c)** An ordered blend of lactose and salbutamol harvested from Asmasal Clickhaler.

Another approach for improving DPI efficiency that emerged in the 1990s was developing particle engineering technologies. Edwards et al.^(93)^ demonstrated that particles with geometric diameters ranging from 5 to 30 μm could be efficiently delivered to the lung if they had very low particle densities since the resulting aerodynamic diameter of the particle was within the respirable range (i.e., less than 5 μm). They observed that highly irregular particles with relatively large geometric diameters dispersed readily due to limited surface contact points with other particles, thus enabling efficient delivery to the lung from even simple DPI devices. They obtained highly irregular particles of drug and poly(lactic acid-co-glycolic acid) or other excipients using an emulsion solvent evaporation technique. Other particle engineering approaches developed for inhalation include the Pulmosphere™ technology developed by Nektar (Fig. 14), spray drying of insulin or other proteins and peptides (Fig. 14), the AIR technology developed by Alkermes, and various technologies utilizing supercritical fluids.

In 2013, Novartis received FDA approval of the Tobi Podhaler for the treatment of lung infections associated with cystic fibrosis (CF). Each dose of Tobi Podhaler consists of inhaling the contents of four capsules that each contain 28 mg of Tobramycin Inhalation Powder (shown in Fig. 14). This product demonstrated that very high doses of powder could be delivered using DPIs, thus expanding the range of diseases that can be treated using DPIs. By 2014, sales of DPIs exceeded $17 billion.^(32)^ At the time of the writing of this article, many DPI products are in development for the treatment of a range of different diseases.

### The emergence of combination products, blockbusters, and modern nebulizer systems

The majority of the drugs currently used in the treatment of asthma and COPD were discovered and/or commercially introduced after 1987. Important new drugs discovered and developed during this period for the treatment of asthma and COPD include new steroids (e.g., budesonide, fluticasone, flunisolide, mometasone), LABAs (e.g., formoterol, salmeterol, indacaterol, vilanterol), and improved anticholinergic drugs (e.g., ipratropium, tiotropium, glycopyrrolate, aclidinium). Research demonstrating the therapeutic synergy of LABAs and corticosteroids^(94,95)^ led to rapid growth in combination therapies. A few of these therapies, in particular Advair/Seretide (MDI and DPI), Spiriva Handihaler DPI, and Symbicort (MDI and DPI), achieved dominant positions in the market accounting for 50% of the market sales in 2014.^(32)^ By 2014, eight inhaler therapies had achieved sales in excess of one billion U.S. dollars (Table 4).

**Table 4.** A List of Inhaler Products with More Than One Billion U.S. Dollars in 2014 Sales^(32)^

*Product*	*Dosage form*	*Company*	*2014 sales ($U.S.)*
Seretide/Advair Diskus	DPI	GSK	7.00 billion
Spiriva Handihaler	DPI	Boehringer Ingelheim	5.07 billion
Symbicort	MDI	Astra Zeneca	2.21 billion
Symbicort Turbuhaler	DPI	Astra Zeneca	1.93 billion
Seretide/Advair HFA	MDI	GSK	1.63 billion
Proair	MDI	Teva	1.45 billion
Flovent/Flixotide	MDI	GSK	1.39 billion
Ventolin	MDI	GSK	1.13 billion

DPI, dry powder inhaler; MDI, metered dose inhaler.

Numerous modern nebulizer systems have been developed to address some of the limitations of conventional air jet nebulizers, in particular the long treatment time and inefficient utilization of drug. Open-vent air jet nebulizers, such as Sidestream^®^ from Philips Respironics, include a vent that increases the total airflow and dose emitted, thus reducing the total time required for dosing.^(58,96)^ Breath-enhanced nebulizers, such as the LC^®^ Plus nebulizer from PARI and Ventstream^®^ from Philips Respironics, include a one-way valve that closes as the patient exhales to minimize the amount of drug emitted to surrounding environment.^(58,96)^ The Pari LC^®^ plus was shown to result in twice the dose delivered to the patient while having a shorter dosing duration than conventional air jet nebulizers.^(97)^

Vibrating mesh nebulizers, such as the AeroNeb^®^ Pro from Aerogen and the eFlow^®^ from PARI, generate fine aerosols without requiring baffles by means of a vibrating mesh that is in contact with the formulation. When the mesh vibrates, the formulation is atomized through hundreds or thousands of small nozzles contained in the mesh. Vibrating mesh nebulizers tend to have the advantages of faster dosing times, less formulation retained in residual volumes of the system, and smaller device size than air jet nebulizers.^(96)^ Further description of modern nebulizers can be found elsewhere.^(19,58,96)^

A number of compact, multidose aqueous delivery systems have been developed since 1987. These combine the soft respirable aerosol generated by modern tabletop nebulizers with the portability and convenience of MDI and DPI products. Aradigm's AERx inhaler, developed in the 1990s, used sterile formulation stored in individually packaged blister strips that contained a layer with an array of laser-drilled holes. When the patient inhaled, pressure was applied to the blister and the liquid formulation was atomized through the laser-drilled holes into a fine spray capable of efficiently being delivered to the lung.^(98)^ The AERx device reached clinical testing with a number of drugs, but has not yet been commercialized. Aqueous delivery systems using vibrating meshes (Aerogen's AeroDose^®^ device), electrospray (Battelle's Mystic™ device), and a number of other approaches have been brought to varying stages of development.^(98)^

**FIG. 15.**
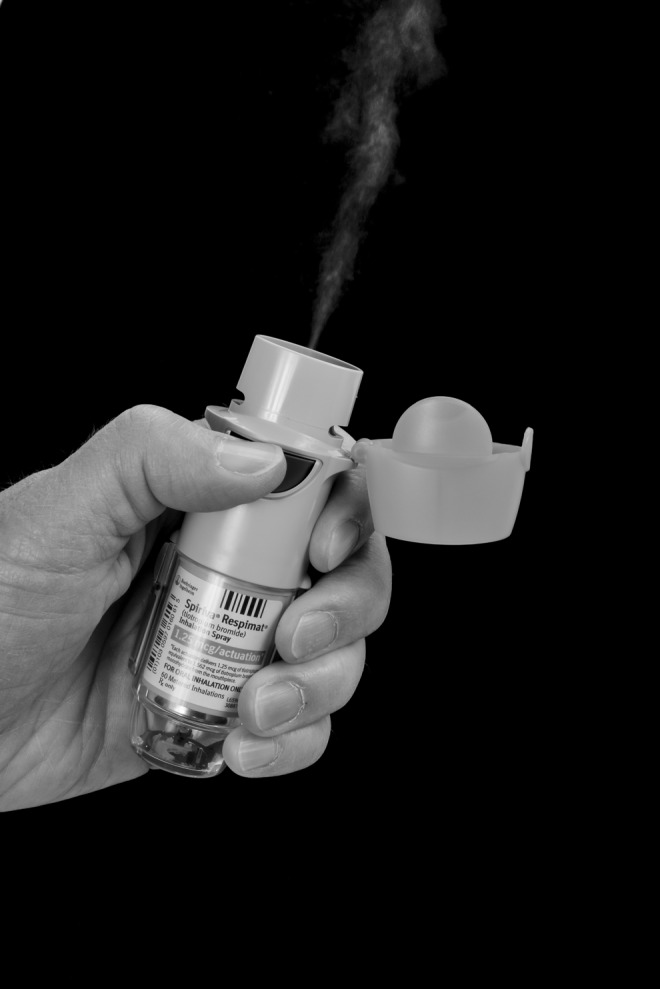
A picture of a Respimat inhaler.

The first commercially available compact aqueous delivery system was the Respimat^®^ Inhalation Spray system (Fig. 15) developed by Boehringer Ingelheim and approved in Germany in 2004 and in the United States and elsewhere shortly thereafter. Respimat has a reservoir containing up to 120 doses of formulation. Before dosing, the patient twists the base of the device that meters out a dose and compresses a spring, which then serves as the energy source to deliver the formulation. When the patient inhales, they press a button that releases the spring and forces the formulation through a complex nozzle configuration to generate a respirable aerosol.^(98,99)^ Multiple products have been approved using the technology, including Spiriva Respimat (tiotropium), Combivent Respimat (ipratropium bromide and albuterol), Striverdi Respimat (olodaterol), and Stiolto™ Respimat (tiotropium bromide and olodaterol).

### The trials and tribulations of inhaled insulin

The concept of utilizing the lung as a portal of entry to deliver insulin and other proteins and peptides dates back to an early inhaled insulin clinical trial in 1925.^(100)^ However, beginning in the late 1980s, there was a significant increase in research and development in the area of aerosol delivery of insulin and other proteins and peptides. By the end of the 1990s, more than 20 large molecules had been evaluated in human clinical dosing^(101,102)^ with inhaled insulin leading the wave of development. It was estimated in 1994 that ∼5% of the U.S. population had diabetes with associated healthcare costs of $40 billion.^(103)^ Insulin injections available at the time were effective when used correctly, but patient compliance was hindered by fear of injections and social stigmas associating needle injections and drug use, as well as the inconvenience of needing to refrigerate injectable insulin products.^(104)^ These shortcomings of insulin injections provided a market opportunity for inhaled insulin.

In the early 1990s, a number of systems were developed to commercialize inhaled insulin products. Inhale Therapeutics (which later became Nektar) began developing the Exubera DPI using a spray drying process to generate human insulin powder that was stored in individual blisters. Alkermes developed an alternate DPI approach using their AIR^®^ insulin particle engineering technology to generate powder consisting of highly porous particles that were delivered using a DPI device that was simpler and smaller than the Exubera device. An advantage of both DPI approaches was that the particle engineering produced stabilized powders that could be stored without refrigeration.

Aradigm developed the AERx^®^ Insulin Diabetes Management System (iDMS). The AERx iDMS was a battery-powered portable nebulizer system that aerosolized insulin contained in a liquid formulation. The electronic capabilities of this system provided helpful guidance to the patient on the correct usage of the device. A disadvantage of this system was that the blisters containing the liquid insulin formulation needed to be refrigerated. All three systems advanced into late-phase clinical testing led by Nektar's Exubera.

Nektar, along with partner Pfizer, conducted extensive clinical testing of Exubera during the late 1990s and early 2000s, including a number of studies required to assess the potential impact of inhaled insulin on lung function, to characterize comparative effectiveness among smokers and nonsmokers and assessing the safety and efficacy among patients with asthma and COPD.^(105)^ After well over a decade of investment and development, Nektar and Pfizer obtained approval of Exubera from the FDA in January 2006, although on the condition of continuing extensive clinical trials to further understand the effect of inhaled insulin on the lung.^(106)^

Despite projections that the product would eventually reach sales on the order of $2 billion,^(104)^ Exubera sales were slow from the start. A number of factors are reported to have contributed to this,^(104,105,107)^ including (1) insurance companies were reluctant to pay premium pricing on a product that did not have a clear clinical benefit; (2) the additional ongoing monitoring of lung function created inconvenience and further expense for the patient; (3) the large device did not fit the discrete dosage form that diabetics desired; (4) achieving the correct dosage may have been confusing based on labeling of doses in milligrams rather than units of insulin; (5) by the time Exubera reached the market, the pain associated with insulin injections had been significantly reduced by smaller sharper needles; and (6) concern about lung cancer was raised when the FDA reported a higher incidence rate of new primary lung cancer for patients treated with Exubera in the clinic compared with patients not treated with Exubera, although the FDA noted that there were too few cases to know if the lung cancer was related to Exubera.

In 2007, Pfizer removed Exubera from the marketplace after a disappointing $12 million in product sales in the first three quarters of 2007.^(107)^ Shortly after the removal of Exubera from the market, Novo Nordisk terminated its collaborative development with Aradigm of the AERx iDMS and Eli Lily terminated its collaborative development with Alkermes of the AIR insulin system even though both systems were in Phase III clinical trials.^(105)^

MannKind, a late entry into the inhaled insulin field, continued development of their DPI product using their Technosphere's engineered powder formulation and achieved approval from the FDA for Afrezza^®^ in 2014. Afrezza was launched by Sanofi in partnership with MannKind in February of 2015, but Sanofi terminated their license and collaboration agreement in January of 2016 after sales of €5 million (∼$6 million) in the first 9 months of 2015. MannKind announced their intention to continue marketing Afrezza while seeking a different marketing partner. Whereas in the 1990s, the lung was often viewed as a portal of entry to systemic circulation for a broad range of therapies,^(101,102,108)^ the limited commercial success of Exubera and Afrezza has cast a cloud of uncertainty not only on the future of inhaled insulin but also on the future of inhaled therapeutic proteins and peptides in general.

## Diseases Currently Being Treated Using Therapeutic Aerosol Delivery

In spite of the challenges associated with the commercialization of inhaled insulin, therapeutic aerosol delivery remains an attractive option for treating conditions beyond asthma and COPD. Inhalation therapy is a mainstay of CF treatment. Ninety percent of CF patients die from lung destruction associated with chronic lung infections with Pseudomonas aeruginosa being a particularly problematic pathogen.^(109)^ Inhaled antibiotics are a cornerstone of CF treatment due to the ability to achieve high concentrations of the antibiotic in the lung while minimizing systemic exposure. Important antibiotics for treating CF lung infections include colistin, tobramycin, and aztreonam lysine. Due to the high dose of antibiotic to be delivered, inhaled antibiotics have traditionally been delivered through a nebulizer.^(109)^ However, recent high-dose DPIs with highly dispersible engineered powders (e.g., TOBI^®^ Podhaler^®^ from Novartis and Colobreathe^®^ from Forest Laboratories, Inc.) have proven to be effective treatments for CF.

In addition to treating infections, inhalation therapy is also used to help break down the viscous mucus layer associated with CF.^(109–111)^ Nebulized dornase alfa (Pulmozyme developed by Genentec) was approved by the FDA in 1993 and it reduces sputum viscosity and results in improved airway clearance. Nebulized hypertonic saline and DPI delivery of mannitol have been developed to improve airway hydration, which may result in improved mucociliary clearance.^(109–111)^ Excellent summaries of the treatment of CF through therapeutic aerosol delivery can be found elsewhere.^(109–111)^

Therapeutic aerosols have been developed to treat numerous other diseases. Relenza, developed by GSK and commercialized in 1999 for treatment of influenza caused by influenza A and B viruses, uses a modified Diskhaler DPI to deliver zanamivir. Zanamivir reduces the duration and severity of symptoms from influenza by binding to the active site of the neuraminidase protein, rendering the influenza virus unable to escape its host cell and infect others.^(112)^ Flumist, developed by MedImmune and approved by the FDA in 2003, delivers live attenuated influenza vaccine taken in a nasal spray.

Nasal delivery of live influenza virus proved more effective than inactivated vaccine delivered intradermally.^(113)^ Aerosol delivery of measles and measles-rubella vaccines offer the potential for superior immune response compared with injected vaccines.^(114)^ An additional potential benefit of inhaled vaccines is the possibility to avoid the cold chain requirement of many vaccines, which is particularly problematic for delivering vaccinations in developing countries. Highly dispersible engineered powders with room temperature stability could be delivered with simple disposable DPIs and offer significant advantages for vaccine delivery in developing countries.^(115)^

Miacalcin nasal spray, commercialized by Novartis, delivers the peptide salmon calcitonin for treatment of osteoporosis. Nebulized iloprost and epoprostenol have been demonstrated to be effective at treating severe pulmonary hypertension.^(116)^ Controversial e-cigarettes, first commercialized in 2004 for delivery of nicotine for smoking cessation purposes, reached $6 billion in sales in 2015,^(117)^ but FDA regulations announced in May of 2016^(118)^ may cut into these sales. Therapeutic aerosols are being investigated for treating other diseases as well.

## Looking to the Future

There are many factors that may shape the future therapeutic aerosol delivery market, including healthcare cost pressures and increasing technical capabilities available for use in inhalation delivery systems. With healthcare costs in the United States projected to increase to 20% of GDP (gross domestic product) by 2020,^(119)^ there is increasing pressure to expand the availability of generic pharmaceutical products. The expansion of access to healthcare in emerging market countries will lead to a further emphasis on generic inhalers.

The looming expiration of patent protection on numerous blockbuster therapies has resulted in significant investment in the development of generic inhalers and much debate and regional differences in terms of the regulatory requirements needed to demonstrate the bioequivalence of generic inhalers.^(120–123)^ In 2013, the FDA provided some clarity on the agency's regulatory expectation for generic applications through two Draft Guidance publications.^(124,125)^ At the time of the writing of this article, there are no generic HFA MDI products available on the U.S. market and there is one generic MDI approved in Europe—Sirdupla™, a generic of Seretide^®^ Evohaler^®^ developed by Mylan and 3 M. There are currently no approved generic DPIs in either the United States or Europe, although a number are known to be in development, including a generic of Advair for which Mylan has filed an ANDA with the FDA.

It is likely that the push for low-cost inhalers will be countered by a desire for new high-tech inhalers with enhanced capabilities. A number of high-tech add-on devices for MDI, DPI, and nebulizer products have been commercialized for MDI products (MD Turbo developed by Respirics, Propeller developed by Propeller Health, Smartinhaler developed by Adherium Ltd.).^(126)^ Benefits to the patients of some of these products include providing reminders to take a dose or order a new inhaler, providing breath actuation, training the patient on the appropriate inhalation maneuver, and recording the time and location of each dose to evaluate adherence to the prescribed dosing regimen.

Some systems, such as the system offered by Propeller Health, have Bluetooth functionality to sync with apps on mobile devices and allow the patient to share treatment data with others so that family members or physicians can monitor adherence or even control of the disease state. The data that can be generated using high-tech systems are of value for policy makers and insurance companies to make informed decisions on public health matters (such as identifying asthma hotspots) and coverage of pharmaceutical products,^(127)^ but privacy issues will undoubtedly need to be addressed. In addition to add-on systems, MDIs and DPIs are being developed with data capabilities already built into the device. If such systems can be demonstrated to improve pharmacoeconomic outcomes, they may gain widespread market acceptance in spite of their higher cost and substantially change the interaction between the patient and their inhaler in the future.

## Conclusion

Therapeutic aerosol delivery has been a primary means of treating lung conditions, particularly asthma, for more than 3500 years. Over this entire period, atropine and related compounds have played an important role in therapeutic aerosol delivery and remain crucial in the treatment of lung diseases today. In ancient times, therapeutic aerosols were often delivered by smoking or placing herbal mixtures in a heated container and inhaling the resulting vapor. Advances in manufacturing capabilities at the dawn of the Industrial Revolution led to more sophisticated techniques for generating therapeutic vapors or nebulizing medicated solutions. The Industrial Revolution led to a shift from therapeutic aerosols being custom produced by someone directly associated with the care of the patient to devices being produced by a person or company completely unassociated with the patient.

The first MDI was introduced in 1956 and dramatically advanced the landscape of therapeutic aerosol delivery. The first DPI was developed in the mid-19th century, but DPIs did not gain market prominence until the 1990s. The signing of the Montreal Protocol in 1987 led to a surge in innovation in inhaler development that has shaped the current inhaler market. In the future, high-tech solutions to improve drug delivery and patient compliance will likely lead to market acceptance of smart inhalers. However, the desire for high-tech inhalers will be countered by the increasing healthcare cost pressures and will likely ensure that MDI and DPI therapies remain important components of therapeutic aerosol delivery.
